# Dolphin Morbillivirus in Eurasian Otters, Italy

**DOI:** 10.3201/eid2502.180256

**Published:** 2019-02

**Authors:** Iolanda Padalino, Giovanni Di Guardo, Antonio Carbone, Pasquale Troiano, Antonio Parisi, Domenico Galante, Maria Assunta Cafiero, Marta Caruso, Lucia Palazzo, Laura Guarino, Laura De Riso, Cinzia Centelleghe, Sandro Mazzariol, Antonio Petrella

**Affiliations:** Istituto Zooprofilattico Sperimentale della Puglia e della Basilicata, Foggia, Italy (I. Padalino, A. Carbone, P. Troiano, A. Parisi, D. Galante, M.A. Cafiero, A. Petrella);; University of Teramo, Teramo, Italy (G. Di Guardo);; Istituto Zooprofilattico Sperimentale della Puglia e della Basilicata, Matera, Italy (M. Caruso);; Istituto Zooprofilattico Sperimentale della Puglia e della Basilicata, Potenza, Italy (L. Palazzo);; Istituto Zooprofilattico Sperimentale della Puglia e della Basilicata, Taranto, Italy (L. Guarino);; Ente Parco Nazionale del Cilento, Salerno, Italy (L. De Riso);; University of Padua, Padua, Italy (C. Centelleghe, S. Mazzariol)

**Keywords:** dolphin morbillivirus, *Morbillivirus*, Eurasian otter, *Lutra lutra*, infection, host range, Mediterranean, Italy, viruses

## Abstract

We report biomolecular evidence of dolphin morbillivirus in 4 wild Eurasian otters (*Lutra lutra*) from southern Italy; 2 animals showed simultaneous immunohistochemical reactivity against morbilliviral antigen. These cases add further concern and support to the progressively expanding host range of dolphin morbillivirus in the western Mediterranean Sea.

The genus *Morbillivirus* comprises several lympho-epithelio-neurotropic, highly pathogenic RNA viruses of domestic and wild vertebrates, including aquatic mammals. Among them, cetacean morbillivirus (CeMV) has been responsible since the 1980s for dramatic epidemics in free-ranging cetaceans worldwide (*1*). Specifically, the CeMV strain termed dolphin morbillivirus (DMV) has caused at least 4 unusual mortality events (UMEs) among striped dolphins (*Stenella coeruleoalba*) in the western Mediterranean Sea and, to a lesser extent, long-finned pilot whales (*Globicephala melas*) and other wild cetaceans from the same region (*1**–**3*).

We report evidence of DMV infection in 4 wild Eurasian otters (*Lutra lutra*) from southern Italy (Apulia and Basilicata regions). The animals, all adult females, belonged to a group of 7 individuals found dead at Parco Nazionale del Cilento, a large national park that extends to the coastline of southwestern Italy. The animals underwent necropsy at Istituto Zooprofilattico Sperimentale della Puglia e della Basilicata (Foggia, Italy) during 2016 and 2017, according to an official agreement between the institute and the park aimed at assessing the health and conservation status of the otter population.

Within a multidisciplinary approach framework, we conducted in-depth histopathologic, microbiologic, parasitologic, and ecotoxicologic analyses on the 7 otters, along with biomolecular (reverse transcription PCR [RT-PCR]) and immunohistochemical (IHC) investigations for *Morbillivirus* spp. After using a technique amplifying a highly conserved fragment of the *Morbillivirus* nucleoprotein (NP) gene (*4*), we applied 2 additional methods aimed at detecting DMV-specific hemagglutinin (HA) (*5*) and NP gene sequences (*6*) for more detailed analysis. To increase the biomolecular results’ reliability, we performed all the extraction, amplification, and sequencing steps in 3 different laboratories. We also conducted the histopathological and IHC analyses in 3 different laboratories. For IHC analysis, we used a commercial monoclonal antibody (VMRD, https://www.vmrd.com) against the NP antigen of canine distemper virus (CDV), including adequate morbillivirus-positive and -negative control tissues in each run.

At necropsy, we found multiple traumatic injuries, probably caused by motor vehicles and deemed the putative cause of death, in all 7 otters. Microscopically, we observed a bilateral, subacute-to-chronic, broncho- or bronchiolo-interstitial pneumonia, showing endobronchial, endobronchiolar, or endoalveolar macrophage infiltration, thickening of alveolar septa, and lympho-histiocytic septal infiltration, in 2 otters; 1 also showed a multifocal, portal and lobular, nonsuppurative hepatitis. Lungs and kidneys from these 2 animals were molecularly positive for DMV (Figure, panel A). Their pulmonary tissue showed positive morbilliviral antigen immunostaining in vascular walls and endothelial cells, as well as in alveolar epithelial cells (consistent, or not, with hyperplastic type II pneumocytes); we also found positive immunostaining in thickened alveolar septa and endoalveolar macrophages (Figure, panel B). Although the advanced postmortem autolysis did not enable us to obtain reliable histopathological information from the 5 remaining otters, biomolecular analyses yielded positive results from several tissues (submandibular lymph node, parotidal gland, lung, brain, heart, kidney, urinary bladder, and liver) in 2 of them (Figure, panel A).

**Figure:**
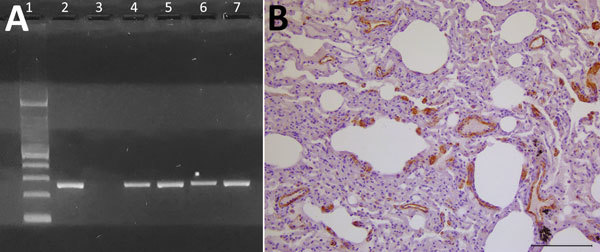
Evidence of dolphin morbillivirus infection in Eurasian otters (*Lutra lutra*), southwestern Italy. A) Comparison of nucleoprotein gene amplification products from infected otters, obtained by reverse transcription PCR. A specific band at the expected molecular weight of 287 bp is shown. Lane 1, molecular weight marker (Tracklt 100bp DNA Ladder; Invitrogen, http://www.thermofisher.com); lane 2, positive control (lung tissue from an infected striped dolphin, *Stenella coeruleoalba*); lane 3, negative control (distilled water); lanes 4–7, samples from morbillivirus-positive Eurasian otters: LL-290, lung (lane 4); LL-291, kidney (lane 5); LL-3380, lung (lane 6); LL-7318, lung (lane 7). B) Mayer’s hematoxylin counterstain of lung tissue shows marked and widespread immunohistochemical labeling for morbillivirus antigen (dark areas), particularly evident at the level of vascular walls and endothelial cells and, to a lesser extent, of alveolar epithelial cells (morphologically consistent, or not, with hyperplastic type II pneumocytes) as well as of thickened alveolar septa. Scale bar indicates 100 μm.

Sequencing of all the 200-bp NP gene viral amplicons obtained (GenBank accession nos. MG836265–8) showed remarkable differences from the available CDV sequences but high homology (99%) with 2 DMV sequences (GenBank accession nos. EF469546.1, KU720625.1). We compared an additional 456-bp HA gene fragment (GenBank provisional accession no. MG905831), obtained from the kidney of 1 of the otters we tested, with 2 DMV isolates recovered from Mediterranean striped dolphins in 2007; this fragment displayed 100% homology with 1 DMV isolate (GenBank accession no. HQ829973) and 99.56% homology with the other (accession no. AJ608288).

Susceptibility to morbilliviruses is not new to *L. lutra* otters; CDV infection has been documented, with subsequent risk for viral spillover to coastal pinnipeds (*7*). In this respect, the endemic behavior and the progressively expanding host range of DMV in the western Mediterranean Sea are a matter of concern (*2**,**3**,**8*), as exemplified by a peculiar case of infection in a captive harbor seal (*Phoca vitulina*), a species with a mixed marine–terrestrial ecology (*9*).

How the otters in this study may have acquired DMV infection is unknown. Of note, 8 cases of DMV infection were detected during 2016 and 2017 in dolphins stranded along the Ionian Sea coast, in an area geographically consistent with that in which the 4 DMV-infected otters were found. It is possible that >1 dolphins with DMV-associated brain lesions could have entered the rivers or lagoons inhabited by the otters and transmitted the virus to them. Alternatively, the otters’ movement toward and placement close to the sea, clearly documented for 1 of them (A. Petrella, unpub. data), could underlie a subsequent encounter with DMV-infected dolphins. As an additional or complementary option, susceptible invertebrate hosts might have acted as DMV reservoirs, similarly to what has been reported for *Baicalia carinata* and *Limnaea auricularia* mollusks, which probably were a source of infection during the CDV epidemic among Baikal seals (*Pusa sibirica*) in 1987 (*10*).

In conclusion, we identified DMV infection among Eurasian otters in southwestern Italy, along the coast of the western Mediterranean Sea. The effect of DMV infection on the health and conservation of the threatened Eurasian otter populations warrants further investigation.
